# Acute COPD exacerbation treatment with noninvasive ventilation

**DOI:** 10.1038/s41598-023-33871-z

**Published:** 2023-04-21

**Authors:** Ewert Ralf, Alexander Heine, Anne Obst, Karoline Koerner, Veit Hustig-Kittler, Michael Boesche, Mohamed Elhadad, Beate Stubbe, Michael Westhoff

**Affiliations:** 1 Division of Pneumology, Department of Internal Medicine B, University Hospital Greifswald, F.-Sauerbruchstrasse, 17489 Greifswald, Germany; 2 Center for Pneumology and Thoracic Surgery, Lung Clinic Hemer, Hemer, Germany; 3 Witten/Herdecke University, Witten, Germany

**Keywords:** Chronic obstructive pulmonary disease, Signs and symptoms

## Abstract

The establishment of a guideline for long-term noninvasive ventilation treatment (LTH-NIV) of acute hypercapnic exacerbations of chronic obstructive pulmonary disease (AECOPD) requiring acute ventilation has proven elusive. Most studies thus far have shown no mortality benefit of long-term noninvasive ventilation treatment. Using retrospective analysis of the data of our patients (n = 143) recruited from 2012 to 2019, we aimed to compare patients discharged with and without long-term noninvasive ventilation. The follow-up results showed no significant difference (p = 0.233) between the groups [LTH-NIV (n = 83); non-NIV (n = 60)] regarding readmission due to clinical worsening. However, the first- and second-year survival rates were 82% and 72%, respectively, in the LTH-NIV group and significantly different (p = 0.023) from 67 and 55% in the non-NIV group. The statistical models showed a significant mortality risk for the non-NIV group, with a hazard ratio (HR) of 2.82 (1.31; 6.03). To the best of our knowledge, this is the first study to demonstrate the mortality benefit of long-term NIV therapy for patients with AECOPD under real-world conditions.

## Introduction

Noninvasive ventilation has been part of the treatment spectrum of acute exacerbations of chronic obstructive pulmonary disease (AECOPD) for years. Its benefits in reducing acidosis, hypercapnia, dyspnoea, and mortality have been demonstrated in prospective studies^1^. In addition, the need for intubation and invasive ventilation could be reduced^2^. It is undisputed that NIV in AECOPD reduces the length of hospital stay and mortality^3^.

The situation regarding NIV as a long-term treatment option following recovery from AECOPD, especially with acute respiratory failure and the need for NIV, is less clear. A recently published Cochrane analysis was only able to examine four randomized studies in detail on this question. In addition to a small improvement in the partial pressures for oxygen and carbon dioxide, there was little to no improvement in the quality of life after 3 and 12 months with NIV therapy^4^. Only the need for hospital readmission was reduced under NIV, but this was not reflected in better survival. A systematic analysis of studies on the initiation of NIV in patients after a recent hypercapnic exacerbation showed no advantage in terms of survival for this group compared to patients without such NIV therapy^5^. Only the inpatient readmissions were reportedly significantly reduced under NIV.

Based on these data, a European group of experts decided to issue a ‘conditional recommendation for the use of LTH-NIV after acute hypercapnic respiratory failure’^6^. The German recommendation on ‘Noninvasive and invasive ventilation as a therapy for chronic respiratory insufficiency—Revision 2017’^7^, which is currently being revised, recommends the initiation of intermittent NIV for COPD patients after AECOPD with respiratory acidosis if hypercapnia (P_a_CO_2_ > 53 mmHg) is still present 14 days after the end of acute ventilation.

Using our patients from 2012 to 2019, we examined how the recommendation to initiate LTH-NIV after AECOPD is reflected in the ‘real world’ of clinical care and which outcomes are related to it.

## Methods

### Patient recruitment, inclusion and exclusion criteria

In total, 151 COPD patients were recruited between January 1st, 2012 and June 30th, 2019 for the current study. Inclusion criteria was treatment with NIV at the Greifswald University Hospital (UMG) due to an acute exacerbation with hypercapnic respiratory failure. The diagnosis of COPD was determined retrospectively through examination of the medical documentation of the patients. The indication for NIV therapy was determined by the emergency physician or a pulmonologist and was based on the current guidelines^8^. The index admission was the first hospitalization day for acute respiratory failure requiring NIV therapy during the abovementioned observation period.

Exclusion criteria were any of the following: preexisting NIV therapy, muscular or neuromuscular diseases involving respiratory muscles, diagnosis of obesity-hypoventilation syndrome, coincident airway or lung diseases other than COPD, unstable coronary artery disease (CHD), known relevant cardiac comorbidity, planned adjustment to intermittent NIV, established NIV in the context of prolonged ventilation weaning, a tracheostomy in connection with the current exacerbation or other uncontrollable comorbidities or palliative therapy concepts.

The patients were then divided into two groups:

- *Group 1* included 83 patients discharged with home long-term noninvasive ventilation (LTH-NIV) and
- *Group 2* included 68 patients discharged without LTH-NIV initiation (non-NIV).

Eight patients from Group 2 received LTH-NIV during the follow-up period. The switch to LTH-NIV took place on average 8 months after the day of discharge. These patients were excluded from further analyses (per protocol analysis with 60 patients).

The basic goal of acute NIV in AECOPD was to reduce dyspnoea and normalize the blood gases, especially P_a_CO_2_ (target < 50 mmHg) and HCO_3_^−^ (target < 30 mmol/L). With progressive clinical stabilization, ventilation pauses were established to attempt ‘NIV weaning’. All patients with an increase in P_a_CO_2_ during NIV pauses and/or increasing dyspnoea were offered LTH-NIV.

According to the German guidelines on prolonged weaning patients received repeated and intensive training in using NIV and the interface during the hospital stay^7^. During hospitalization at least 3 blood gas analyses were performed on daily routine during NIV-therapy. Due to the parameter results NIV-parameters were adjusted accordingly.

An adaptation and optimization phase, training in the use of NIV therapy at home and mask training for the patients resulted in a significantly longer ventilation time.

#### Methodology

Patients with AECOPD and the need for NIV treatment were retrospectively identified using the medical documentation of cases of the Department of Internal Medicine at the Greifswald University Hospital that were designated under the category ‘ventilation’. The data acquisition was limited to the period from January 1st, 2012 to June 30th, 2019. The patients’ data were extracted by analysing the medical discharge letters, in particular regarding previous illnesses, their medical history and data from previous inpatient stays. The following patient-relevant data were extracted: case number, date of birth, age, sex, height, weight, COPD stage, smoking status, date of inpatient admission and discharge, index of quality of life (according to ECOG), comorbidities (according to the Charlson index) and treatment-associated data (selected laboratory values, vital signs, and ventilation parameters).

The intensive care patient documentation program ‘Integrated Company Manager’ (ICM, Draeger Comp., Lübeck) used at the Greifswald University Hospital, the laboratory management software ‘Lauris’ and the clinical workstation system ‘Klinische Arbeitsplatzsystem’ (KAS) served as databases for this purpose.

#### Follow-up observations

**Figure 1:**
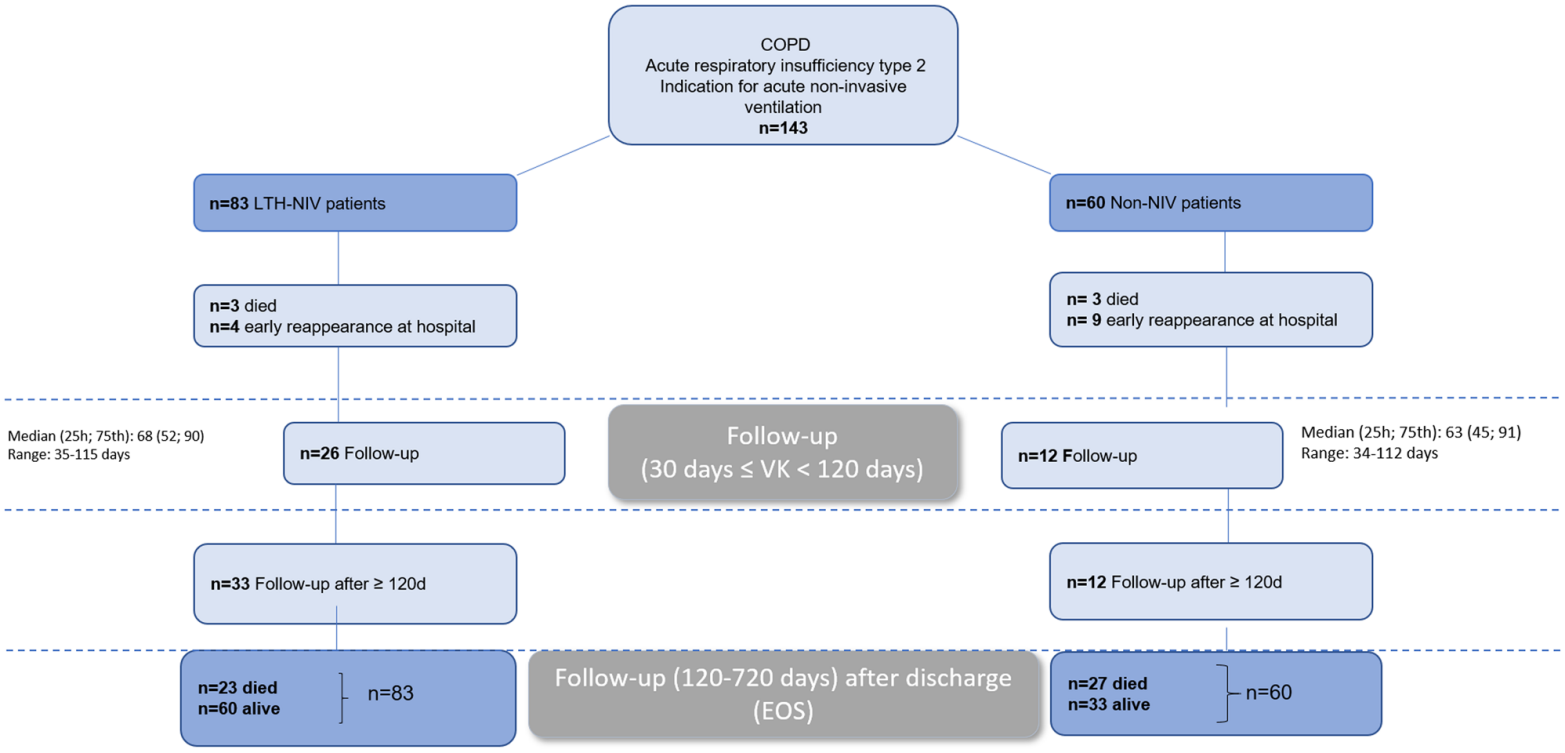
Flow chart of the study design.

We followed up the patients using the ‘digital case files’ in the KAS from the time of discharge from the hospital onwards. The case files contain all inpatient and outpatient stays in the Greifswald University Hospital, as well as the associated diagnoses and doctor's letters. During a follow-up period of two years (from the day of discharge), we recorded both the point in time and the circumstances of repeated outpatient or inpatient treatments. Moreover, we scheduled a first check-up for all patients approximately 8–12 weeks after discharge in our pneumology outpatient clinic (Fig. 1).

Furthermore, the date of death was documented from the case files. If the date of last contact in the case file was after the two-year observation point, the patient was considered alive at the end of the two years in the statistical analyses. In all other cases, telephone contact was made with the patient, the relatives, or the general practitioner to obtain information on the status of the patient. The primary endpoint was death within 2 years of follow-up.

#### Data acquisition/statistics

For the description of the data, we summarized continuous variables as the median (25% percentile, 75% percentile) or mean (± standard deviation, SD) and categorical variables as absolute frequencies and percentages. Differences between the two groups were tested using the Mann–Whitney U test and the Chi^2^ test. The Wilcoxon signed-rank test was used to check whether there were differences between the blood gas tests at the time of discharge and at the time of follow-up. To investigate the connection between LTH-NIV/non-NIV and mortality, we applied a Kaplan–Meier survival curve and compared it using a log-rank test. In addition, Cox regression analyses were performed to estimate the influence of the selected parameters on mortality with hazard ratios (HRs) and 95% confidence intervals (CIs) reported.

All analyses were performed using SAS program 9.4 (SAS Institute Inc., Cary, NC, USA).

Before the start of data collection, the study protocol was submitted to the UMG ethics committee and was approved under the number BB038/19 after an internal review process.

The check-ups were terminated after 8–12 weeks because, as a specialized pneumology centre, we serve a large area (radius of 150 km), and outpatient follow-up visits are quite time-consuming.

### Ethics approval

The study was approved by the Ethics Committee of Greifswald University under the number No BB038/19 of April 05, 2019. The study was carried out in accordance with the requirements of the Declaration of Helsinki and the local Ethics Committee.

### Consent to participate

The patients gave written informed consent for the study participation.

## Results

The baseline characteristics are shown in Table 1 and Supplement Table S1. BMI was significantly lower in the non-NIV patients (p < 0.001). A BMI > 30 was present in 46 of the 83 patients in the LTH-NIV group and 19 of the 60 patients in the non-NIV group. BMI did not show a significant influence on our analysed follow-up data (data not shown). Regarding the secondary diagnoses, there were significant differences, with chronic heart failure, chronic kidney disease and diabetes mellitus showing significantly lower prevalences in the non-NIV group. The grading of the comorbidities according to the Charlson index thus showed a significantly higher disease burden in the LTH-NIV patients.

**Table 1:** Patient characteristics.

Parameter	Total	LTH-NIV	Non-NIV	p-value
n	143	83	60	
Age [years]	69 (60; 76);	68 (59; 76)	69 (62; 78)	0.088
Females	54 (37.8%)	32 (38.6%)	22 (36.7%)	0.818
Height [cm]	170 (165; 175);	170 (165; 175)	170 (165; 175)	0.977
Weight [kg]	85 (70; 105);	90 (75; 114)	75 (65; 100)	0.002
Body mass index [m^2^/kg]	28.0 (24.2; 36.7)	32.6 (25.5; 38.7)	26.0 (22.0; 31.4)	< 0.001
Smoking habits (known) (n)	117	64	53	0.577
Active smoker	50 (42.7%)	27 (42.2%)	23 (43.4%)	
Ex smoker	55 (47.0%)	32 (50.0%)	23 (43.4%)	
Never smoker	12 (10.3%)	5 (7.8%)	7 (13.2%)	
GOLD-stage (known) (n)	n = 131	n = 83	n = 48	0.943
GOLD-stage I/II	35 (26.7%)	22 (26.5%)	13 (27.1%)	
GOLD-stage III/IV	96 (73.3%)	61 (73.5%)	35 (72.9%)	
Charlson Comorbidity Index				0.037
None or mild (level 0 0 points)	0	0	0	
Mild to moderate (level 1 1–2 points)	56 (39.1%)	26 (31.3%)	30 (50.0%)	
Moderate to severe (level 2 3–4 points)	56 (39.1%)	34 (41.0%)	22 (36.7%)	
Severe (level 3 ≥ 5 points)	31 (21.7%)	23 (27.7%)	8 (13.3%)	
Index of physical limitation (Eastern Co-operative Oncology Group)	138	78	60	0.437
No limitations	2 (1.4%)	2 (2.6%)	0	
Minor limitations	66 (47.8%)	40 (51.3%)	26 (43.3%)	
Self-supporter not able to work	58 (42.0%)	29 (37.2%)	29 (48.3%)	
In need of care 50% bedridden during the daytime	11 (8.0%)	6 (7.7%)	5 (8.3%)	
Critically ill completely disabled	1 (0.7%)	1 (1.3%)	0	

The chosen peak and end-expiratory pressure was significantly higher in the LTH-NIV group (IPAP 19 (15; 22); PEEP 6 (5; 8)) than in the non-NIV group (IPAP 15 (10; 18); PEEP 5 (4; 6)). The resulting tidal volumes were not significantly different in either group. In the LTH-NIV group, 86.8% (72/83) of the patients and in the non-NIV group, 68.3% (41/60) of the patients received additional oxygen administration during their hospital stay (Table 2).

**Table 2:** Blood gas findings, and NIV modes/parameters during hospitalization.

Parameter	Total	LTH-NIV	Non-NIV	p-value
n	143	83	60	
pH	7.35 (7.28; 7.42)	7.37 (7.28; 7.43)	7.34 (7.29; 7.41)	0.467
P_a_O_2_ [mmHg]	65.8 (55.3; 80.6)	61.3 (54.3; 74.6)	68.0 (56.3; 89.4)	0.014
P_a_CO_2_ [mmHg]	62.1 (52.4; 74.7)	64.4 (56.4; 76.0)	58.1 (46.2; 71.5)	0.011
HCO_3_- [mmol/L]	30.3 (26.9; 34.8)	32.9 (29.3; 37.5)	27.8 (25.9; 30.2)	< 0.001
Base excess [mmol/L]	n = 141 6.0 (3.0; 10.7)	n = 81 8.9 (5.8; 12.9)	4.0 (1.7; 6.1)	< 0.001
Lactate [mmol/L]	n = 141 1.0 (0.7; 1.4)	n = 81 0.9 (0.6; 1.4)	1.0 (0.7; 1.7)	0.189
Initial NIV-parameter				
Modus	139	83	56	< 0.001
Controlled modus	28 (20.1%)	10 (12.0%)	18 (32.1%)	
Controlled-assisted modus	78 (56.1%)	63 (75.9%)	15 (26.8%)	
Assisted-spontaneous modus	33 (23.7%)	10 (12.0%)	23 (40.1%)	
Peak pressure (inspiratory) in mbar	n = 141 17 (14; 20)	19 (15; 22)	n = 58 15 (10; 18)	< 0.001
Peak end-expiratory pressure (PEEP) in mbar	n = 141 6 (5; 7)	6 (5; 8)	n = 58 5 (4; 6)	< 0.001
Tidal volume [ml]	n = 140 579 (454; 718)	550 (447; 712)	n = 57 617 (472; 775)	0.121
Minute volume [l]	n = 140 11.3 (8.9; 14.5)	10.2 (8.1; 12.4)	n = 57 14.2 (11.0; 17.0)	< 0.001
Blood gas analyses before dismissal				
n	134	78	56	
pH	7.44 (7.41; 7.47)	7.44 (7.41; 7.46)	7.45 (7.42; 7.47)	0.222
P_a_O_2_ [mmHg]	65.9 (57.6; 72.0)	66.0 (58.2; 71.8)	65.7 (57.4; 72.4)	0.980
P_a_CO_2_ [mmHg]	43.2 (40.4; 47.3)	43.0 (40.4; 47.7)	43.6 (40.3; 46.5)	0.896
HCO_3_^−^ [mmol/L]	28.9 (26.7; 30.9)	28.7 (26.5; 30.8)	29.1 (26.7; 31.1)	0.601
Base excess [mmol/L]	n = 130 5.1 (2.6; 7.0)	n = 74 4.9 (2.5; 7.0)	5.4 (2.8; 7.3)	0.549
Lactate [mmol/L]	n = 133 1.0 (0.7; 1.4)	n = 77 1.0 (0.7; 1.3)	1.0 (0.7; 1.6)	0.243

PaO_2_ partial pressure O_2_; P_a_CO_2_ partial pressure CO_2_; HCO_3_^−^ standard bicarbonate.

The mean ventilation time was 21 ± 12 days (median 19 days) in the LTH-NIV group and 6 ± 5 days (median 4 days) in the non-NIV group.

The median length of hospital stay was 15 (10; 24) days [19 (12;30) days in the LT-NIV group and 12 (9;17) in the non-NIV group], with significant differences (p < 0.001) between the two groups.

At the initiation of NIV, all patients showed type II (hypercapnic) respiratory failure, with predominantly mild to moderate acute respiratory acidosis (pH: 7.30–7.35). NIV was initiated with an oronasal mask in different modes, with most of the LTH-NIV group using a controlled-assisted mode (76%) and the majority of the non-NIV group using an assisted-spontaneous mode (41%).

At the time of admission (except for 6 patients) blood-gas analysis was determined under oxygen supply. In most cases the last BGA before discharge was with NIV and oxygen supply (in 30 patients only NIV). The BGA during follow-up (120–720 days) was performed without NIV.

### Follow-up examination

Differences between both groups were evident even before the follow-up visit that was predetermined 6–8 weeks after discharge. In the LTH-NIV group, 3 (3.6%) patients had already died, and another 4 (4.8%) patients were hospitalized due to clinical deterioration. In the non-NIV group, 3 (5.0%) patients died, and an additional 9 (15.0%) required hospitalization due to clinical deterioration (Fig. 1).

Of the LTH-NIV patients, 26 received a follow-up visit at our facility after a median of 68 (52; 90) days. In the non-NIV group, a median of 12 patients presented for control after 63 (45; 91) days.

Up to the end of the observation (2 years after initial discharge), 33 patients in the LTH-NIV group and 12 patients in the non-NIV group accepted the offer of an additional consultation.

**Figure 2:**
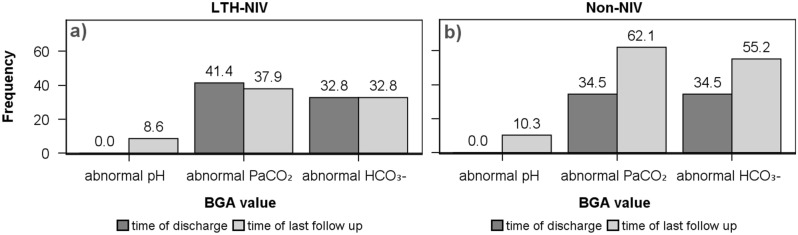
Comparison of selected BGA-values between LTH-NIV patients (**a**) and Non-NIV patients (**b**).

At the follow-up visit (n = 38) and at the end of the study (n = 87), 58 of them from the LTH-NIV group), blood gas analyses were carried out as part of the outpatient aftercare under stable clinical conditions. In the LTH-NIV group, no patient showed a pH < 7.35 upon discharge, but a decrease below this value was documented in 8.6% during the observation period (Fig. 2a). Regarding P_a_CO_2_ values, 41.4% still had a pathological value upon discharge (men > 46 mmHg; women > 43 mmHg). The mean value was 44.2 ± 6 mmHg, with six cases > 53 mmHg. The proportion of patients with pathological values changed only slightly over the course of the observation to 37.9%. A standard bicarbonate > 30 mmHg was found in 32.8% of the patients at discharge and during the study’s observation period (Tables 2, 3).

**Table 3:** Blood gas analyses at follow-up.

Parameter	Total	LTH-NIV	Non-NIV	p-value
n	33	23	10	
pH	7.41 (7.37; 7.44)	7.42 (7.37; 7.47)	7.39 (7.35; 7.42)	0.078
P_a_O_2_ [mmHg]	58.7 (48.5; 70.3)	59.4 (48.8; 74.8)	52.4 (47.2; 70.3)	0.378
P_a_CO_2_ [mmHg]	46.7 (40.0; 56.1)	46.1 (39.1; 52.0)	54.1 (42.5; 58.1)	0.085
HCO_3_^−^ [mmol/L]	29.3 (26.8; 33.0)	29.3 (26.6; 32.5)	31.1 (26.8; 33.3)	0.666
Base excess [mmol/L]	4.1 (1.7; 6.5)	4.1 (1.7; 7.0)	3.9 (0.8; 5.8)	0.695
Lactate[mmol/L]	n = 31 1.2 (0.8; 1.9)	n = 21 1.2 (0.8; 1.6)	1.2 (0.8; 2.2)	0.799
Blood gas analyses at follow-up (all maximum one year after dismissal)				
n	87	58	29	
pH	7.42 (7.38; 7.45)	7.42 (7.39; 7.45)	7.39 (7.37; 7.42)	0.007
P_a_O_2_ [mmHg]	59.9 (52.5; 69.9)	60.0 (55.3; 72.9)	54.8 (47.2; 67.1)	0.024
P_a_CO_2_ [mmHg]	44.6 (39.1; 51.9)	43.1 (38.7; 47.6)	49.3 (42.1; 55.3)	0.010
HCO_3_^−^ [mmol/L]	29.0 (25.8; 31.7)	28.2 (25.6; 31.5)	30.5 (27.1; 32.2)	0.099
Base excess [mmol/L]	3.7 (1.0; 5.8)	3.5 (0.9; 6.1)	4.1 (1.6; 5.8)	0.428
Lactate [mmol/L]	n = 81 1.3 (0.9; 2.0)	n = 53 1.4 (1.0; 2.0)	n = 28 1.2 (0.8; 1.9)	0.438

PaO_2_ partial pressure O_2_; P_a_CO_2_ partial pressure CO_2_; HCO_3_^−^ standard bicarbonate.

In the non-NIV group, none of the patients had a pH < 7.35 at discharge, but 10.3% of the patients showed lower values over time (Fig. 2b). The proportion of pathological P_a_CO_2_ values at discharge was 34.5% and at the end of the study was 62.1%. At discharge, the mean CO_2_ partial pressure was 44.0 ± 5 mmHg, with two patients in this group having values > 53 mmHg. A standard bicarbonate > 30 mmHg was found in 34.5% of the patients at discharge and 55.2% at the last follow-up examination.

**Figure 3:**
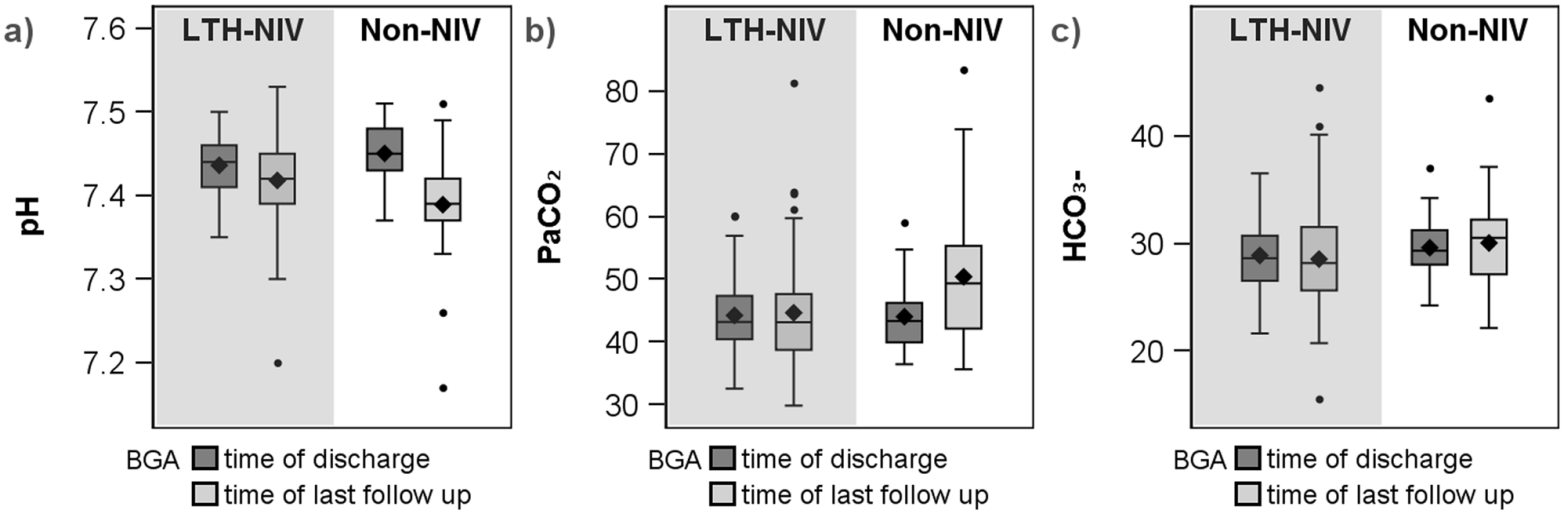
Comparison of pH (**a**), paCO_2_ (**b**) and HCO_3_^−^ (**c**) between LTH-NIV patients and Non-NIV patients depending on the time of discharge or last follow-up.

Comparisons between the selected parameters of the blood gas analyses (BGA) at the time of discharge and the last follow-up are shown in Fig. 3. The LTH-NIV group showed a significant drop in pH values (p = 0.044) and no significant differences in P_a_CO_2_ (p = 0.574) or HCO_3_^−^ (p = 0.230). The non-NIV group showed a significant drop in pH (p < 0.001) and a significant increase in P_a_CO_2_ values (p = 0.001), but no significant difference in bicarbonate values (0.471).

The need for hospital readmission in the event of clinical deterioration showed no significant (p = 0.233) difference between the two groups during the two-year observation period.

### Survival

**Figure 4:**
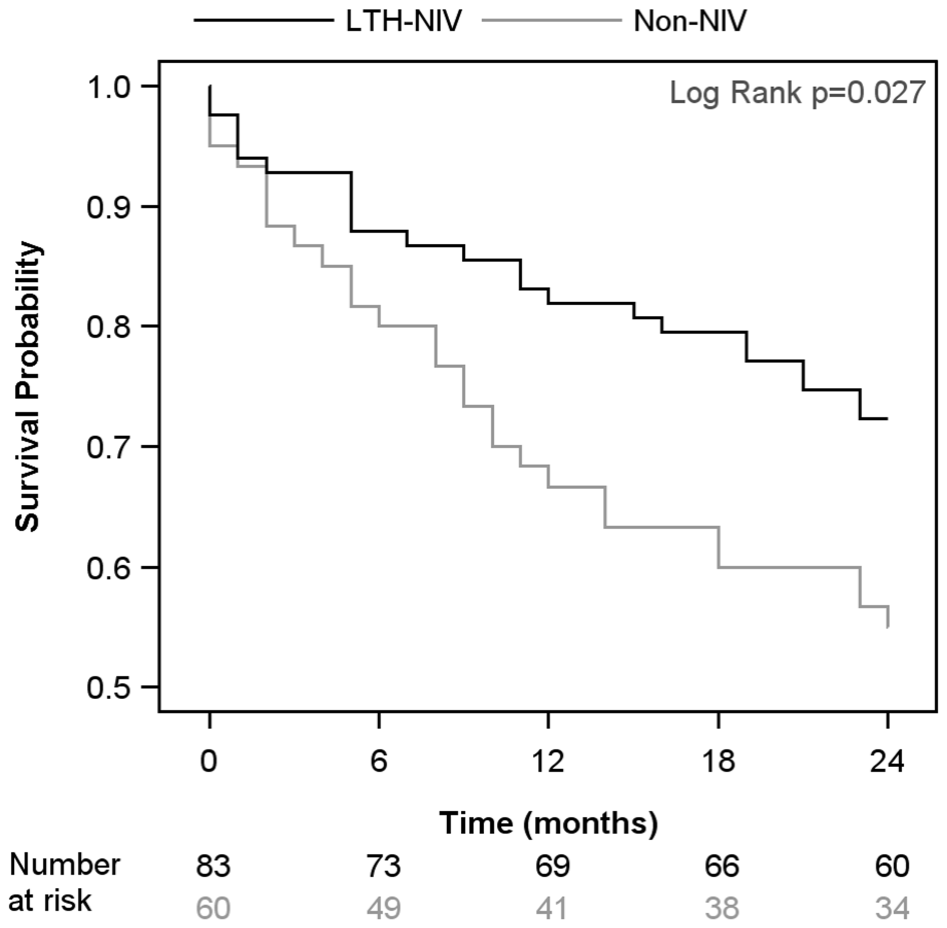
Comparison of survival curves between LTH-NIV patients and Non-NIV patients.

During the observation period, 23 out of 83 (27.7%) of the patients in the LTH-NIV group and 27 out of 60 (45.0%) of the patients in the non-NIV group died (Fig. 4). Accordingly, the 1- and 2-year survival rates in the LTH-NIV group were 82% and 72% vs. 67% and 55% in the non-NIV group and they were significantly different (p = 0.027).

### Predictors of premature death

The Kaplan–Meier curve shows a shorter survival time in the non-NIV group (Fig. 4). This result could be confirmed using unadjusted Cox regression models (Supplement Table S2). Non-NIV-discharged patients had a 1.8-fold mortality risk increase [HR: 1.84 (1.06; 3.22)], with men showing a 1.9-fold mortality risk increase [HR: 1.91 (1.01; 3. 59)]. Furthermore, there was a positive association with age [HR per increase of 1 year: 1.04 (1.01; 1.07)] and the presence of gastric ulcer disease [HR: 5.71 (1.75; 18.6)]. Compared to ECOG stages 0/1, ECOG stage 2 was associated with a 2.9-fold mortality risk increase [HR: 2.86 (1.50; 5.46)], and ECOG stages 3/4 were associated with a 5.6-fold mortality risk increase [HR: 5.55 (2.32; 13.27)].

Concerning parameters of the BGA recorded at follow-up, patients with acidosis had a fourfold higher mortality risk [HR 3.93 (1.58; 9.77)], and patients with pathologically increased P_a_CO_2_ had a 2.5 times higher risk of dying [HR: 2.51 (1.16; 5.45)].

Additional adjustment for age, sex, and ventilation time confirmed the increased risk of mortality for patients discharged without NIV [HR: 2.82 (1.31; 6.03)] (Table 4). The inclusion of P_a_CO_2_ from the follow-up examination as a confounder in the Cox regression model (Subgroup b) led to a slightly higher estimate for the mortality risk [HR: 3.09 (1.16; 8.23)].

**Table 4:** Two different models for the calculation of predictive variables for survival in multivariate Cox analysis.

	HR (95% CI)	p-value
Age (years)	1.04 (1.01; 1.07)	0.010
Model 1* (Patients (n = 143), Events (n = 50))		
Male (ref.: Female)	2.19 (1.15; 4.15)	0.017
Non-NIV (ref.: LTH-NIV)	2.82 (1.31; 6.03)	0.008
Ventilation time (days)	1.03 (1.01; 1.06)	0.018
Model 2^# (^Patients (n = 87), Events (n = 28))		
Age (years)	1.06 (1.02; 1.11)	0.004
Male (ref: Female)	1.85 (0.15; 4.27)	0.152
Non-NIV (ref: LTH-NIV)	3.09 (1.16; 8.23)	0.024
Ventilation time (days)	1.04 (1.00; 1.07)	0.039
P_a_CO_2_ (ref: female P_a_ CO_2_ ≤ 43 mmHg and male ≤ 46 mmHg)	2.74 (1.23; 6.13)	0.014

*HR* hazard ratio, *CI* confidence interval.

*Model 1 included age, gender, ventilation therapy, and ventilation time.

^#^Model 2 included age, gender, ventilation therapy, and ventilation time, P_a_CO_2_.

## Discussion

In a retrospective data analysis of 143 COPD patients with a need for NIV during AECOPD, we were able to show that 1) LTH-NIV did not significantly reduce the need for hospital readmission during a two-year follow-up (p = 0.233) and 2) patients with LTH-NIV after discharge had a significantly better 2-year survival (p = 0.027). These results were confirmed by Cox regression analysis.

Comparable studies on the initiation of LTH-NIV after AECOPD are limited and they all follow different methodological concepts. In one study, all patients (n = 38) were treated with NIV for six months after AECOPD, and the remaining patients (n = 26) were then randomized into an NIV and a control group. After twelve months, 15% in the NIV vs. 77% of the control group showed a clinical deterioration (in 7 out of 13, NIV had to be initiated again for AECOPD)^10^. The authors concluded that ‘patients who had previously required mechanical ventilation for treatment of acute respiratory failure and who remain hypercapnic thereafter may benefit from long-term NIV’. Another study^11^ randomized AECOPD patients who remained clinically stable without NIV for 48 h into the LTH-NIV group (n = 24; PaCO_2_ 58 mmHg) vs. the CPAP group (n = 23; paCO2 55 mmHg). After one year of follow-up, the LTH-NIV group showed a significant reduction in AECOPD but with high drop-out rates.

The RESCUE trial included 201 AECOPD patients who were randomized very early (> 48 h without ventilation and PaCO_2_ > 45 mmHg). After one year, no effect could be seen in terms of reduced exacerbations or survival in patients with LTH-NIV compared to conservative therapy alone^12^. However, many of those treated with NIV in the acute phase had a pH > 7.35 and therefore had no indication for NIV therapy.

A different methodological approach was applied to 116 patients after AECOPD^13^. In this prospective study, patients with persistent hypercapnia (P_a_CO_2_ > 53 mmHg) 2–4 weeks after termination of NIV initiated because of respiratory acidosis were randomized to LTH-NIV + LOT or only LOT. Subsequently, 59 patients received long-term oxygen therapy (LOT) vs. 57 LTH-NIV + LOT. The results showed a significant reduction in the time to readmission or death in the LHT-NIV + LOT group vs. LOT therapy alone. The 12-month risk of readmission or death was 63.4% in the LHT-NIV + LOT group vs. 80.4% in the LOT group, resulting in an absolute risk reduction of 17.0% (95% CI, 0.1–34.0%). At 12 months, 16 out of 57 (28.1%) patients had died in the LHT-NIV + LOT group vs. 19 out of 59 (32.2%) in the LOT group.

The age of our patients, 67 ± 10 years, compared well with other studies (67 ± 9.6 in Murphy et al.^13^; 64 ± 8.6 in Struik et al.^12^; the LTH-NIV group in Cheung et al.^11^ 70 ± 7.8 years; the NIV group in Funk et al.^10^ 62 ± 6 years). The proportion of female patients, 38% in our study, was lower than in other studies (53% in (Murphy et al.^13^); 59% Struik et al.^12^; 56% (Funk et al.^10^) and significantly lower than 9% (Cheung et al.^11^). At 33 ± 11.2, the BMI of our LTH-NIV patients was significantly higher than in the studies considered for comparison (25 ± 5.4 in the NIV group in Struik et al.^12^; median 22 (18.8–2.5) of the NIV group in Murphy et al.^13^; 19 ± 3.6 in the NIV group in Cheung et al.^11^; 24 ± 4.3 in the NIV group in Funk et al.^10^. A closer look at our patients makes it clear that 46 out of 83 patients in the LTH-NIV group and 16 out of 60 patients in the non-NIV group had a BMI > 30. Thus, at least some of our patients met the criteria for the classification of COPD obesity overlap syndrome.

Regarding the presence of comorbidities, in the study by Funk et al.^10^, 15% of the patients in the NIV group had diabetes mellitus. This is comparable to our data. However, cardiovascular comorbidities were more frequent in our patients. This might influence the comparability of the studies.

The length of inpatient stay during the index event (start of observation) was reported to be 13–14 days by Cheung^11^. Our patients had a median length of inpatient stay of 15 days.

While the factors influencing the in-house mortality of patients requiring NIV in AECOPD have been rigorously investigated^14,15^, little is known about their long-term course. Sprooten et al. showed that older age, a longer hospital stay and a poor response to NIV are independent risk factors for 2-year mortality^9^. Moreover, multiple studies have indicated a worse long-term prognosis in male patients after an episode of NIV in AECOPD^16,17^. In a study with 574 patients (357 men, mean age 68 ± 11 years), with a median observation of 27 months, significant influencing factors of long-term mortality (at 1 year, 30%) identified in the univariate analysis were older age, higher Charlson score, lower baseline levels of haematocrit and albumin, and a lower pH level after 24 h. Multivariate analysis yielded only older age and a low albumin level as significant factors influencing long-term mortality^18^.

In line with previous studies, our analyses showed a significantly lower mortality among women^16,17^. We were able to confirm a previously reported increased mortality associated with increasing age^9,18^ and more extensive comorbidity^18^. In addition, we were able to show that our patients had a significantly lower survival rate if they had greater functional limitations, a more pronounced acidosis on admission to the hospital, and a persistently increased PaCO_2_ value during the outpatient follow-up observation. Although we could not find comparable data from the literature, our data are supported by observing the significant impact on reduced survival when there is an inadequate response to NIV therapy^9^. On the other hand, successful LTH-NIV is associated with a reduction in paCO_2_ values and a drop in bicarbonate concentration^19^.

In our multivariate analysis, a longer ventilation time during the hospital stay was significantly associated with increased mortality, which is in line with our previously reported results of patients with prolonged weaning^20^.

The 1-year mortality in our study was 18% for the LTH-NIV patients and 33% for the non-NIV patients. These values were thus comparable to those of the prospective study of 116 patients by Murphy et al.^13^ (28% in the NIV + LOT group and 32% in the non-NIV group). Comparably, other studies also show a mortality rate of 30% in the first year after AECOPD without NIV^18^.

### Limitations

Because the data were obtained retrospectively, not all clinical and laboratory data for the patients were available. No information was recorded on the initial response to NIV (normalization of pH > 7.35; decrease of P_a_CO_2_ < 6.0 kPa (45 mmHg); good tolerance to NIV and no clinical requirement for intubation)^9^. The routine check-ups were not continued after the initial check-up for patients with AECOPD discharged from hospital since a large area is served by the specialized pneumology center and outpatient follow-up visits are pretty time-consuming. Due to the fact that there were no therapy dropouts the compliance was excellent.

Questions about the indications for LTH-NIV are gaining in importance, driven by the continuously increasing number of patients receiving LTH-NIV in Germany (17,958 new appointments in 2019, Schwarz SB et al. Pneumologie 2021^21^). While there is a clear recommendation for the initiation of LTH-NIV after successful weaning from long-term ventilation, further investigations are certainly required to determine the indication in patients after hypercapnic AECOPD with respiratory acidosis. In our retrospective study, taking confounders under account, we were able to demonstrate a significant influence of NIV treatment on the survival of patients receiving long-term noninvasive ventilation over a period of two years after an acute exacerbation of COPD. To the best of our knowledge, we were therefore for the first time able to prove a positive effect of NIV in this group of patients under ‘real world conditions’.

## Supplementary Information

Supplementary Table 1.

Supplementary Table 2.

## Data Availability

Data are available by contacting our statistician Dr. Anne Obst (anne.obst@med.uni-greifswald.de).

## Abbreviations

LTH-NIV: Long-term home noninvasive ventilation therapy
AECOPD: Acute exacerbation of chronic obstructive pulmonary disease
HR: Hazard ratio
NIV: Noninvasive ventilation
COPD: Chronic obstructive pulmonary disease
CHD: Chronic heart disease
P_a_CO_2_: Carbon dioxide partial pressure
HCO_3_^−^: Standard bicarbonate
ECOG: Eastern Cooperative Oncology Group
ICM: Intermediate Care Medicine
KAS: Klinisches Arbeitsplatzsystem
SD: Standard deviation
CI: Confidence interval
UMG: University Medicine Greifswald
IPAP: Inspiratory positive airway pressure
PEEP: Positive endexpiratory pressure
BGA: Blood gas analysis
BMI: Body mass index
LOT: Longterm oxygen therapy
