# Supplementary material for: Acute COPD exacerbation treatment with noninvasive ventilation
Source: Sci Rep. 2023 Apr 21;13:6586. doi: 10.1038/s41598-023-33871-z (PMC10121675; doi:10.1038/s41598-023-33871-z)
Supplement: Supplementary file 1 — Supplementary Table 1. [file 41598_2023_33871_MOESM1_ESM.docx]

Supplement Table 1: Comorbidities of included patients.

| Parameter | **Total** | | **LTH-NIV** | | **Non-NIV** | | | **p-value** | |
| --- | --- | --- | --- | --- | --- | --- | --- | --- | --- |
| Comorbidities | |  | |  | |  |  | |  |
| Chronic lung disease | | 143 (100%) | | 83 (100%) | | 60 (100%) | 1.000 | |  |
| Heart failure | | 69 (48.3%) | | 47 (56.6%) | | 22 (36.7%) | 0.018 | |  |
| Kidney disease | | 37 (25.9%) | | 29 (34.9%) | | 8 (13.3%) | 0.004 | |  |
| Mild elevation of liver enzymes | | 41 (28.7%) | | 28 (33.7%) | | 13 (21.7%) | 0.115 | |  |
| Diabetes mellitus | | 60 (42.0%) | | 44 (53.0%) | | 16 (26.7%) | 0.002 | |  |
| Diabtes mellitus with end organ damage | | 22 (15.4%) | | 18 (21.7%) | | 4 (6.7%) | 0.014 | |  |
| Myocardial infarction | | 20 (14.0%) | | 10 (12.0%) | | 10 (16.7%) | 0.432 | |  |
| Peripheral vascular disease | | 22 (15.4%) | | 10 (12.0%) | | 12 (20.0%) | 0.193 | |  |
| Cerebrovascular disease | | 11 (7.3%) | | 5 (6.0%) | | 6 (8.8%) | 0.544 | |  |
| Dementia | | 4 (2.8%) | | 3 (3.6%) | | 1 (1.7%) | 0.486 | |  |
| Ulcer disease | | 3 (2.1%) | | 3 (3.6%) | | 0 | 0.264 | |  |
| Severe elevation of liver enzymes | | 1 (0.7%) | | 1 (1.2%) | | 0 | 0.394 | |  |
