# Supplementary material for: Acute COPD exacerbation treatment with noninvasive ventilation
Source: Sci Rep. 2023 Apr 21;13:6586. doi: 10.1038/s41598-023-33871-z (PMC10121675; doi:10.1038/s41598-023-33871-z)
Supplement: Supplementary file 2 — Supplementary Table 2. [file 41598_2023_33871_MOESM2_ESM.docx]

**Supplement Table 2: Unadjusted Cox-Regression Models.**

| **Parameter** | **HR** | **ChiSq** | **ProbChiSq** | **Total** | **Event** |
| --- | --- | --- | --- | --- | --- |
| time_day_admission | 1.02 (1.00; 1.03) | 2.80 | 0.094 | 143 | 50 |
| **Age** | **1.04 (1.01; 1.07)** | **6.41** | **0.011** | **143** | **50** |
| Height | 1.01 (0.97; 1.04) | 0.12 | 0.724 | 143 | 50 |
| Weight | 0.99 (0.98; 1.00) | 2.44 | 0.118 | 143 | 50 |
| Body mass index | 0.98 (0.95; 1.01) | 2.49 | 0.114 | 143 | 50 |
| pH | 0.53 (0.03; 10.4) | 0.17 | 0.677 | 143 | 50 |
| pO_2_ | 1.01 (1.00; 1.02) | 2.06 | 0.151 | 143 | 50 |
| pCO_2_ | 1.00 (0.98; 1.02) | 0.00 | 0.947 | 143 | 50 |
| HCO_3_ | 0.99 (0.94; 1.03) | 0.30 | 0.584 | 143 | 50 |
| Base excess | 0.99 (0.94; 1.04) | 0.29 | 0.588 | 141 | 50 |
| Peak pressure | 0.98 (0.93; 1.03) | 0.55 | 0.457 | 141 | 49 |
| Peak end-expiratory pressure | 0.98 (0.84; 1.15) | 0.04 | 0.844 | 141 | 49 |
| VT | 1.00 (1.00; 1.00) | 0.03 | 0.859 | 140 | 49 |
| MV | 1.01 (0.94; 1.08) | 0.06 | 0.808 | 140 | 49 |
| Last_peak pressure | 0.98 (0.92; 1.04) | 0.59 | 0.442 | 141 | 49 |
| Last_PEEP_EPAP | 0.93 (0.81; 1.07) | 1.00 | 0.318 | 141 | 49 |
| VT_1 | 1.00 (1.00; 1.00) | 0.12 | 0.727 | 139 | 49 |
| MV_1 | 1.04 (0.97; 1.11) | 1.11 | 0.292 | 139 | 49 |
| Ventilation time_in_days | 1.00 (0.98; 1.02) | 0.03 | 0.871 | 143 | 50 |
| **Dismissal Non-NIV (ref.: LTH-NIV)** | **1.84 (1.06; 3.22)** | **4.64** | **0.031** | **143** | **50** |
| Charlson_Index_Level | 1.32 (0.93; 1.89) | 2.39 | 0.122 | 143 | 50 |
| **Female** | **1.91 (1.01; 3.59)** | **4.40** | **0.036** | **143** | **50** |
| First Re-admission | 1.28 (0.71; 2.30) | 0.71 | 0.399 | 143 | 50 |
| COPD_level | 1.38 (0.68; 2.78) | 0.85 | 0.358 | 131 | 45 |
| Smoking habit_m | 1.29 (0.77; 2.14) | 0.99 | 0.321 | 117 | 37 |
| **ECOG (ref.: no or mild limitation)** |  |  |  | **138** | **49** |
| **Self-supporter, not able to work** | **2.86 (1.50; 5,46)** | **10.13** | **0.002** |  |  |
| **In need of care, 50% bedridden during daytime or critically ill, disabled** | **5.55 (2.32; 13.27)** | **14.83** | **< 0.001** |  |  |
| Myocardial infarction | 0.97 (0.44; 2.16) | 0.01 | 0.943 | 143 | 50 |
| Heart failure | 1.61 (0.92; 2.82) | 2.80 | 0.094 | 143 | 50 |
| Peripheral vascular disease | 1.23 (0.60; 2.54) | 0.31 | 0.577 | 143 | 50 |
| Apoplex | 2.37 (1.06; 5.27) | 3.62 | 0.057 | 143 | 50 |
| Dementia | 3.53 (1.09; 11.4) | 3.16 | 0.075 | 143 | 50 |
| **Ulcer disease** | **5.71 (1.75; 18.6)** | **5.33** | **0.021** | **143** | **50** |
| Diabetes mellitus | 0.67 (0.37; 1.20) | 1.90 | 0.168 | 143 | 50 |
| Kidney disease | 1.32 (0.72; 2.42) | 0.78 | 0.378 | 143 | 50 |
| Elevated liver enzymes | 0.97 (0.53; 1.81) | 0.01 | 0.935 | 143 | 50 |
| Diabetes with end organ damage | 1.03 (0.70; 1.50) | 0.02 | 0.884 | 143 | 50 |
| Severe liver disease | 1.69 (0.87; 3.28) | 1.53 | 0.216 | 143 | 50 |
| Modus | 0.79 (0.56; 1.11) | 1.78 | 0.182 | 139 | 49 |
| last_BGA_pCO_2_ | 1.03 (0.97; 1.09) | 0.82 | 0.366 | 87 | 28 |
| last_BGA_HCO_3_ | 1.03 (0.90; 1.16) | 0.15 | 0.700 | 87 | 28 |
| last_BGA_BE | 1.02 (0.91; 1.15) | 0.12 | 0.726 | 84 | 28 |
| last_BGA_Lactate | 1.08 (0.54; 2.16) | 0.05 | 0.818 | 86 | 28 |
| **FU_pH** | **0.00 (0.00; 0.02)** | **10.47** | **0.001** | **87** | **28** |
| **FU_pH_dy** | **3.93 (1.58; 9.77)** | **6.61** | **0.010** | **87** | **28** |
| FU_pO_2_ | 0.98 (0.96; 1.01) | 2.42 | 0.120 | 87 | 28 |
| **FU_pCO_2_** | **1.05 (1.02; 1.08)** | **7.97** | **0.005** | **87** | **28** |
| **FU_pCO_2__dy** | **2.51 (1.16; 5.45)** | **5.76** | **0.016** | **87** | **28** |
| FU_HCO_3_ | 1.04 (0.96; 1.12) | 0.97 | 0.324 | 87 | 28 |
| FU_HCO_3__dy | 1.91 (0.91; 4.01) | 2.90 | 0.089 | 87 | 28 |
| FU_BE | 1.01 (0.92; 1.10) | 0.03 | 0.873 | 87 | 28 |
| Fu_Lactate | 0.78 (0.43; 1.40) | 0.71 | 0.401 | 81 | 27 |

BE, base excess; BGA, blood gas analyses; COPD, chronic obstructive pulmonary disease; ECOG, Eastern Co-operative Oncology Group; EPAP, expiratory positive airway pressure; HCO_3_, standard bicarbonate; HR, hazard ratio; MV, minute volume; pCO_2_, partial pressure CO_2_; PEEP, peak end-expiratory pressure; pO_2_, partial pressure O_2_; VT, tidal volume; FU, follow-up
